# Interventions addressing the impostor phenomenon: a scoping review

**DOI:** 10.3389/fpsyg.2024.1360540

**Published:** 2024-03-28

**Authors:** Emma Para, Philippe Dubreuil, Paule Miquelon, Charles Martin-Krumm

**Affiliations:** ^1^Department of Psychology, Université du Québec à Trois-Rivières, Trois-Rivières, QC, Canada; ^2^Department of Human Resources Management, Université du Québec à Trois-Rivières, Trois-Rivières, QC, Canada; ^3^Vulnérabilité, Capabilité et Rétablissement (VCR), Ecole de Psychologues Praticiens of Catholic University of Paris, Paris, France; ^4^INSERM INSPIIRE UMR 1319, Lorraine University, Nancy, France

**Keywords:** scoping review, impostor phenomenon, interventions, workplace, adults

## Abstract

**Introduction:**

The Impostor Phenomenon (IP) refers to a psychological experience characterized by unjustified feelings of intellectual and professional fraud, accompanied by the fear of not maintaining performance and of being exposed. IP is receiving increasing attention in the fields of psychological health at work and occupational psychology as well as among the general public, since it affects the functioning of both individuals and organizations. The aim of this scoping review is to map the range of interventions that have been conducted to address IP among individuals experiencing it in a professional context.

**Methods:**

The search and selection process to identify relevant reports was conducted using the PRISMA-ScR methodology and JBI recommendations and resulted in the selection of 31 studies.

**Results:**

The results reported concerning the characteristics of the studies, the interventions described, and the effects identified are heterogeneous. More than half of the studies used research designs (experimental, pre-experimental, exploratory, etc.). Two major types of intervention emerge: training and counseling. The effectiveness of the interventions varies according to the evaluation methodology that was used, although most authors conclude that the proposed intervention is relevant.

**Discussion:**

In light of these results, recognizing and educating individuals regarding the various manifestations of IP, as well as offering support in a group context, appear to be primary intervention levers. Future intervention proposals should explore psychosocial and educational influences as well as the impact of the immediate environment on IP-related beliefs.

## Introduction

Since its emergence in the late 1970s (Clance and Imes, 1978), the concept of impostor phenomenon (IP) is drawing increasing scientific interest and gaining popularity among the general public. It is characterized by an intense and secret psychological experience of intellectual and professional fraud (e.g., Clance and Imes, 1978; Harvey and Katz, 1985). These unjustified feelings of phoniness in the face of success, known as *impostor feelings*, are accompanied by the fear of not being able to reproduce one’s performance and the fear of being discovered (Clance and Imes, 1978; Clance, 1985). IP seems to be a common experience, as Tewfik (2022) pointed out, “*may unwittingly come from common work experiences such as a promotion in which one is suddenly expected to successfully execute unfamiliar responsibilities*” (p. 992). Moreover, going through a major life transition or acquiring a new role, in a professional or a private context, can put individuals at risk of developing IP (Harvey, 1981; Fujie, 2010). IP can also be considered a vicious circle, where each repetition reinforces the dysfunctional beliefs and associated negative feelings (e.g., Clance and Imes, 1978; Clance, 1985; Chrisman et al., 1995). With the exception of rare positive accounts (Fruhan, 2002; McElwee and Yurak, 2010; Tewfik, 2022), the expression of the IP is considered to be very detrimental to health and wellbeing at work (e.g., Vergauwe et al., 2015) and, consequently, to the proper functioning of organizations (e.g., Kets de Vries, 2005). Addressing IP seems to be a significant lever of action for organizations wishing to promote good health at work, and employee fulfillment and performance. Yet, as revealed by the systematic review by Bravata et al. (2020), treatments for IP have been little studied to date. To fill this gap, this review examines interventions to address IP and proposes a benchmark for future contributions to this field of research.

To better understand its objectives, it is worth mentioning the broader context of current knowledge about IP. First of all, data on the prevalence of IP within the population vary widely, from 9% to 82%, depending on the psychometric instrument and cut-off score (Bravata et al., 2020). It is generally associated with a decrease in wellbeing and life satisfaction (Clance and O’Toole, 1987; September et al., 2001). Furthermore, IP can affect individuals’ psychological health in various ways (e.g., Bernard et al., 2002; Brauer and Wolf, 2016). Indeed, it predicts up to 15% of self-reported psychological distress (Oriel et al., 2004). The most frequently associated comorbidities are depression (e.g., Oriel et al., 2004; Leonhardt et al., 2017), anxiety (e.g., Bernard et al., 2002; Kananifar et al., 2015) low self-esteem (Neureiter and Traut-Mattausch, 2016), and somatic symptoms and social dysfunction (Kananifar et al., 2015). Moreover, IP is now considered by many experts to be a psychopathological reality (e.g., Chassangre, 2016) and some researchers have suggested that it should be included in the Diagnostic and Statistical Manual of Mental Disorders (DSM) (Bravata et al., 2020).

Observational studies of IP in a work context have been carried out in various fields, such as academia (e.g., Clance and Imes, 1978; Byrnes and Lester, 1995), healthcare (e.g., Ares, 2018; LaDonna et al., 2018), marketing (e.g., Fried-Buchalter, 1997), and management (Rohrmann et al., 2016). In professional context, IP is generally associated with reduced job satisfaction (Vergauwe et al., 2015). Studies have demonstrated its link with stress (e.g., Rohrmann et al., 2016; Alrayyes et al., 2020), burn-out (e.g., Legassie et al., 2008; Vergauwe et al., 2015), and emotional exhaustion (Leach et al., 2019). Furthermore, IP is known to negatively affect work-life balance (Crawford et al., 2016) and it is positively related to workaholism (Mir and Kamal, 2018), suggesting that persons who experience IP are more likely to work harder and longer, in order to avoid their fear of failure. In addition, it can often become an obstacle to career progression and the motivation to take on a leadership role (Neureiter and Traut-Mattausch, 2016, 2017). It can also influence decisions about whether to continue in an educational program or a career (e.g., Clance and O’Toole, 1987; Blondeau and Awad, 2018). Moreover, IP is usually known to lead to maladaptive organizational behaviors (e.g., Bechtoldt, 2015; Neureiter and Traut-Mattausch, 2016). More recently, Tewfik (2022) has nuanced this observation by demonstrating that those who experience IP were evaluated as more interpersonally effective. What is more, this relational efficiency does not come at the expense of competence-related outcomes. IP is also known as a factor influencing absenteeism and turnover in companies (Kets de Vries, 2005). However, organizational functioning and managerial attitudes can mitigate the deleterious effects of IP (e.g., Bechtoldt, 2015; Crawford et al., 2016).

That being said, an important aspect of the theoretical background is the lack of conceptual clarity regarding IP and the persistent discussion over the nature of the construct. Beyond the lack of uniformity in the terms used to designate it (sometimes a phenomenon, a syndrome, *impostorism*, an experience, a feeling, thoughts, etc.), a lack of consensus remains on its consideration as a trait or a state (Gullifor et al., 2023) or on the dimensionality of the phenomenon (Mak et al., 2019). Indeed, IP was described by early authors as a cognitive, behavioral, and emotional dynamic (Clance, 1985), corresponding to a list of intrapersonal criteria (Clance, 1985; Harvey and Katz, 1985). If, for many years, it was perceived as a stable personality trait (e.g., Topping, 1983; Sonnak and Towell, 2001), it was later seen as an affective state that could manifest itself in certain situations (e.g., Leary et al., 2000; McElwee and Yurak, 2007); a *psychological experience* according to McElwee and Yurak (2010). Since then, this aspect of the discussion has not really been elucidated, as Gullifor et al. (2023) pointed out. In response to this issue, these authors proposed a *trait–state conceptualization of IP.* On the other hand, Mak et al. (2019), in a systematic review of IP measurement scales, identify that the lack of conceptual clarity around the dimensionality of the IP has limited the establishment of a *gold standard measure.*

Despite these critical gaps in the scientific literature, which are legitimately deplored by the authors of systematic reviews on the subject (Mak et al., 2019; Bravata et al., 2020; Gullifor et al., 2023), the field of research of the interventions addressing IP is, quite recently, expanding. According to recent perspectives (e.g., Kark et al., 2022), IP presents as a psycho-social issue that affects the psychological health, wellbeing, and careers of individuals. These findings justify a scoping review, to support both current intervention approaches and the development of future research in this field. A scoping review is particularly relevant to the topic of interventions addressing IP given that the subject is emergent and has not been extensively reviewed. Like systematic reviews, scoping reviews follow a rigorous, systematic literature search and identification process; however, they are more exploratory in nature (Colquhoun et al., 2014; Lockwood and Tricco, 2020).

Therefore, the aim of this scoping review is to provide a more comprehensive overview of the research landscape, map the range of interventions that have been conducted to address IP among individuals experiencing it in a professional context, which is currently unavailable in the literature. In line with this objective, it addresses the following questions: (1) What types of studies and methodologies have been undertaken to explore interventions addressing IP expressed in occupational contexts? (2) What are the modalities and characteristics of these interventions? and (3) What are the effects of these interventions?

## Method

This scoping review uses a systematic approach to analyze and aggregate scientific data on a given topic to identify concepts, theories, sources, and gaps associated with current knowledge (e.g., Munn et al., 2018; Tricco et al., 2018, 2022). However, unlike a systematic review, a scoping review must examine all relevant literature, regardless of the study design. This review follows the Preferred Reporting Items for Systematic Reviews and Meta-Analyses, Extension for Scoping Reviews (PRISMA-ScR) (2018) guidelines and the recommendations of the Joanna Briggs Institute (JBI) (2020). PRISMA-ScR Check List is presented as Supplementary Table 2.

Framing elements were defined to guide the identification and selection of references. These elements concern: (1) the target population of adults in a professional context (such as employees, managers, self-employed workers, but also student trainees and interns), (2) the concept of interventions to address IP, and (3) the organizational, training or private context of these interventions.

### Search and selection process of references

#### Search terms and sources

Relevant references were identified using an iterative process, initiated in November 2022, supported by a librarian specialized in database selection, keyword identification, equation development and search strategy. This approach was then refined in light of the initial results.

Six databases covering psychological, medical, and business literature were selected: APA PsycArticles, APA PsycINFO (EBSCO), Business Source Complete (EBSCO), PubPsych, Scopus, and MEDLINE. The search equation was developed by combining impostor phenomenon (IP) keywords and treatment terms. In the literature, the terms *Impostor Syndrome* (IS) and *Impostor Phenomenon* (IP) are synonymous. Other less common terms, such as *Impostor Experience, Fraud Syndrome*, and *Impostorism*, were also included. These terms were associated with the keywords *Treatment, Intervention, Therapy, Counseling, Rehabilitation*, and *Management*.

The database search identified a total of 394 references. It was subsequently completed by a manual search and consultation of the reference lists, which identified a further 14 references. As an example, the search strategy for APA PsycInfo is presented in Supplementary Table 1.

#### Selection of references

The selection of references was based on predetermined eligibility criteria (Table 1).

**TABLE 1:** Eligibility criteria for reference selection.

Criteria	Inclusion	Exclusion
Population	Adults; Professionals or students working in a professional context (e.g., interns, residents, etc.).	Minors; Non-professional students (i.e., in academic context only).
Exposure	IP in work-related or internship situation.	IP in personal or social context.
Theme or publication subject	Mentions or involves an intervention on IP in work-related or internship situation.	Does not describe the intervention used.
Publication type	Articles in peer-reviewed journals, doctoral theses, chapters in scientific or educational books (e.g., teaching manuals, books for students, professionals, and academics).	Lay literature (e.g., blog articles, non-specialized journals, business reviews, popular or personal development books).
Publication language	English, French.	All others languages.
Publication date	From 1978 (date of emergence of the IP concept) to the day of research.	Before 1978.

According to the JBI, the broad scope of scoping reviews allows for “*less restrictive inclusion criteria*” (Peters et al., 2015, p. 7) and may draw upon data from any type of evidence and research methodology (Peters et al., 2020). Furthermore, based on the defined objectives and framing elements, the definition of inclusion criteria (in terms of dates, diversity of sources and types of methodology) ensured broad coverage of all the literature relevant to this scoping review; including exploratory, prescriptive, and non-experimental scientific literature. In addition, although this research falls within the field of organizational psychology, it was decided to include studies involving students; as long as they are in internship situations, which utilize their skills as future professionals rather than strictly academic competencies. Exclusion criteria were defined to eliminate references that would not have pertinently addressed the questions raised by this scoping review (e.g., excluding studies focusing on children, references without any interventions, etc.).

**FIGURE 1:**
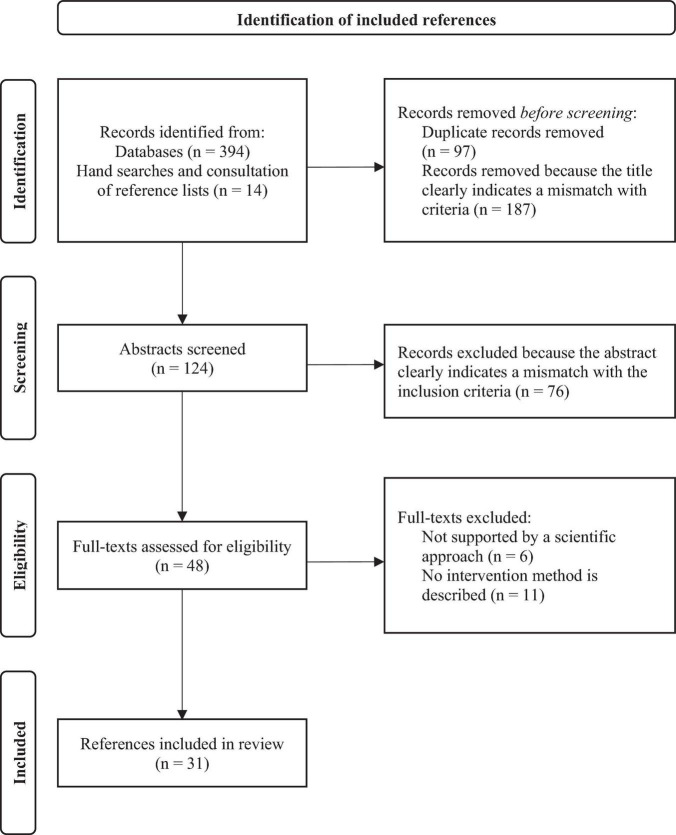
Flow diagram of the selection process of references according to PRISMA-ScR guidelines.

After deleting duplicates, the titles and abstracts of all references were examined for relevance. Those that clearly did not meet the criteria were eliminated. Two reviewers then applied these criteria to all the abstracts retained after the first selection (*n* = 124), and then to the full texts (*n* = 48) to determine their eligibility. In the end, 31 publications were retained. Figure 1 presents the PRISMA flow diagram.

#### Data extraction

The information and characteristics of the selected references were extracted using a coding grid. They included title, authors, geographical origin, year and type of publication, study objectives, study population and sample size (if applicable), participant characteristics, methodology, measures used, type of intervention, results of analyses, and finally, limitations and main conclusions.

## Results

### General overview

Of the 31 references on IP interventions selected, 26 are scientific articles from peer-reviewed journals, with a wide range of contents and formats; 4 are chapters in scientific or professional books and 1 is a doctoral thesis.

The interventions presented in these documents vary depending on the context of the studies. First, most interventions were conducted in Europe or the United States (over 80%). Second, the majority of the interventions were deployed in the healthcare and higher education (university) sectors, among participants reporting IP in relation to their professional activities.

Regarding the characteristics of the participants, most of the interventions were carried out with a sample of participants composed primarily of women. Some even specifically targeted professional women (e.g., Mann et al., 2023). Moreover, in half the studies, the average age of participants was under 40 years. In addition, the fact that the participants were young professionals was sometimes reported as an important aspect (e.g., Harte and McGlade, 2018; Danhauer et al., 2019; Baumann et al., 2020). However, not all authors reported the age of participants (e.g., Deshmukh et al., 2022).

**FIGURE 2:**
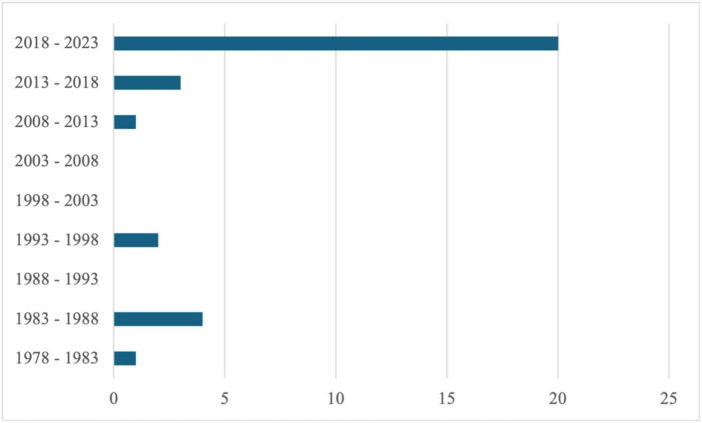
Number of publications related to interventions addressing IP between 1978 and 2023 (October).

In terms of temporal landmarks, it is very interesting to observe that the field of research on interventions addressing IP is recently in full expansion. This is even more true when considering only studies based on a research design (all post-2018). Figure 2 presents the number of publications related to IP interventions over the years. This observation constitutes an additional argument in favor of proposing a synthesis of existing data and gaps in the current literature.

Considering the great heterogeneity of the selected references and to clearly report the results, as well as to be able to make consistent comparisons between types of intervention, the decision was made to split the references into two categories. The objective of splitting the references into two categories is to facilitate the distinction between both and to help the readers further discern the conclusions drawn from these two types of literature. The results from these two types of literature are thus presented separately below.

### Studies carried out using a research design

Table 2 presents references describing an intervention tested using a scientific research design.

**TABLE 2:** References testing an intervention in a scientific study design.

References	Publication type	Study design	Sample size (*N*)	Population type, gender, and age	Variables	Intervention (duration)	Key findings
Baumann et al., 2020	Research article	Cross-sectional (mixed-method)	21	Internal medicine residents Gender not reported (NR) Age NR	*Quantitative* Effects of intervention on resident wellness Learning objectives about IS *Qualitative* Intervention feedback and improvement suggestions	Small-group discussion on the topic of IS, as part of a larger series of discussion sessions on resident wellness (30–45 min)	IS has been linked to resident burnout. After the intervention, 96% of residents felt comfortable recognizing IS in themselves and 62% knew the appropriate next steps after identification. Discussing IS was viewed by 81% of residents as an effective intervention to promote wellness and resiliency.
Chang et al., 2022	Research article	Pre-experimental (mixed-method)	51	Health science students Women (69%), men (31%) Age NR	*Quantitative* Impostor phenomenon Growth mindset Coping strategies *Qualitative* via structured progress reports: value of research, personal growth, and career development	Theory-based workshop on impostor perceptions and growth mindset (90 min)	The workshop enhanced IP awareness and provided coping strategies. Adopting a growth mindset may reduce imposture feelings, explaining the intervention’s effectiveness. Quantitatively, students’ feelings of imposture remained unchanged, but they reported improved management of these feelings and sustained progress in facing their challenges.
Danhauer et al., 2019	Research article	Pre-experimental (quantitative)	65	Early-career women faculty Women (100%) 29 years or younger (6.2%), 30–39 years (75.4%), 40–49 years (18.5%)	*Quantitative* Career knowledge and skills confidence Current perceptions of the work environment Program feedback	Interactive sessions of professional training: *Early Career Development Program for Women* (4 years: 6 sessions of 6–8 h each)	Most skills like *knowing paths to promotion, tailoring communication style, ability to manage conflict and to handle personal–professional role balance* showed pre–post significant improvement. Women reported a significant increase in confidence. A total of 89.3% of participants rated the program as having a very strong impact.
DeCandia Vitoria, 2021	Research article	Case report (qualitative)	1	Psychotherapist Woman Age NR	*Qualitative* Effectiveness of clinical supervision on the IP through confidence, self-empathy, shame, and fear	Individual clinical supervision (narrative therapy and interpersonal neurobiology) (NR)	Through supervision that blended narrative therapy methods with interpersonal neurobiology concepts, the client achieved beneficial results. She regained confidence, saw a reduction in her feelings of shame and fear, and developed self-empathy by creating a new, empowering personal story.
Deshmukh et al., 2022	Research article	Cross-sectional (mixed-method)	30	Clinical radiology faculty (radiologists) Men (63%), women (37%) Age NR	*Quantitative* Impostor phenomenon Burnout Quality rating (post-workshop) *Qualitative* IP personal experience Common representations about IP and burnout	Interactive workshop on IP through medical improvisational techniques (1 h)	A total of 71% of respondents exhibited frequent or intense symptoms of IP. The IP is statistically correlated with burnout within radiologists. Through “*creating an inclusive environment of belonging and acceptance*” a pilot workshop utilizing medical improvisational techniques to address IP was very well received.
Fainstad et al., 2022	Research article	Experimental (randomized controlled trial) (quantitative)	101	Female resident physicians Women (100%) Mean age = 29.4 years (SD = 2.3)	*Quantitative* Impostor phenomenon Burnout Self-compassion Moral injury	Web-based professional group-coaching program: *Better Together Physician Coaching* (6 months) Waiting list control group	In the intervention group, emotional exhaustion decreased by a mean of 3.26 points, IP impostor syndrome decreased by a mean of 1.16 points and self-compassion scores increased by a mean of 5.55 points. An online multiformat group-coaching program may be an effective intervention to decrease burnout and IS and to increase self-compassion.
Gold et al., 2019	Brief research report	Pre-experimental (mixed-method)	30 (18 completed surveys)	Medical students Women (83.3%) Age NR	*Quantitative* Empathy Wellness (interpersonal fulfillment) Loneliness *Qualitative*: groups expectations and goals	Reflection and support groups (6 months: 90 min, biweekly)	For the majority of participants, group sessions enhanced wellbeing, self-awareness, empathy, and fostered a sense of connection. Reflection groups seem to be successful in reducing IS and fostering social belonging.
Haney et al., 2018	Research article	Cross-sectional (mixed-method)	447	Students from 8 healthcare specialties professions Gender NR Age NR	*Quantitative* Impostor phenomenon *Qualitative* Comments about the effects of intervention	Educational inter-professional workshop (1 day)	IP scores are significantly elevated for half of participants and indicate that they have frequent feelings of imposture. As a qualitative outcome, students expressed feelings of liberation and empowerment after the workshop.
Harte and McGlade, 2018	Research article	Exploratory (qualitative)	6	General practice trainees 4 women, 2 men Age NR	*Qualitative* (thematic grouping): General experience of coaching Leadership identity Areas of leadership development	Professional coaching sessions (12 weeks: 6 coaching sessions)	Coaching provides guidance and fosters a sense of identity. It appears to validate clients as leaders and mitigates feelings associated with IP. This approach delivers personalized, in-depth, practical learning.
Liu et al., 2023	Research article	Experimental (randomized controlled trial) (quantitative)	227	College students Cisgender women (77.5%), cisgender men (21.6%), transgender (0.4%), non-binary (0.4%). Mean age = 19.58 years (SD = 2.82)	*Quantitative* Impostor phenomenon Maladaptive perfectionism Psychological distress Fear of self-compassion Core self-evaluation	Brief self-compassion intervention: online training modules (4 × 45 min) No intervention control group	The intervention had significant treatment effects for reducing IP and maladaptive perfectionism. Fear of self-compassion and core self-evaluation appear as moderators of the intervention’s effects but not in the way that was expected.
Magro, 2022	Research article	Descriptive phenomenology (qualitative)	6	Managers (mid-career or senior professionals) Women (100%) Age between 25 and 55 years	*Qualitative* Describe the experience of being coached for impostor syndrome State before coaching Coaching engagement Impact of coaching	Individual professional coaching and group coaching for one participant (NR)	The benefits of coaching about IP are based on “*an emotional process of trusting, learning, uncovering, managing, and sharing*” (p. 68). Through the relationship itself and the tools and techniques learnt, clients are able to improve their awareness and manage their feelings of imposture. However, these effects seem to be only temporary and diminish after the coaching experience is over.
Mann et al., 2023	Research article	Experimental (randomized controlled trial) (quantitative)	1,017	GME women physician trainees Women (100%) Mean age = 30.9 years (SD = 4)	*Quantitative* Impostor syndrome Moral injury Burnout Self-compassion Flourishing	Online group-coaching program: *Better Together Physician Coaching* (4 months) Waiting list control group	Participants had a statistically significant reduction in all scales of burnout, moral injury, and IS, as well as improved self-compassion and flourishing, compared with the control group. Web-based professional group-coaching can improve outcomes of wellbeing and mitigate symptoms of burnout for women physician trainees.
Metz et al., 2020	Research article	Pre-experimental (quantitative)	103	First-year dental students Women (53%), men (47%) 21 years or younger (6%), 22–25 (72%), 25 years or older (22%)	*Quantitative* Impostor phenomenon Student use of the 6 coping mechanisms	Online training module to educate about IP and provide 6 coping mechanisms (6-credit-hour)	There was a reduction in IP scores post-semester, from 63.44 to 59.12. The proportion of students experiencing strong impostor feelings dropped from 13.6% to 4.9%. Common coping strategies included cutting back on non-essential tasks and using scheduling to avoid procrastination. An online training module can enhance IP awareness and assist high-achieving students in managing it.
Ogunyemi et al., 2022	Research article	Retrospective and cross-sectional (quantitative)	198	Medical education cohort Women (77.5%), men (22.5%) 40 years or younger (46.9%)	*Quantitative* Impostor phenomenon Baseline knowledge on IS IS competence subtypes Perception about intervention Post-intervention knowledge of IS	Interactive and reflective educational workshop (60–90 min)	A total of 57% of participants were positive for IS. There was an increase in knowledge survey scores from 4.94 to 5.78 following the intervention. Participants’ recognition of IS as a source of stress, an obstacle to achieving full potential, and a factor in negative relationships or teamwork increased. An interactive workshop focusing on IS can enhance understanding and awareness of the syndrome.
Popovic, 2020	Doctoral dissertation	Interpretative phenomenology (qualitative)	12	Master level marriage and family therapy students 10 women and 2 men Age between 25 and 61 years	*Quantitative* Impostor phenomenon *Qualitative* Levels of differentiation Sense of awareness Anxiety Sense of connectedness Sense of freedom New knowledge	Multimodal intervention including a short psycho-educational training (10–15 min), supervision group (90–120 min), family genogram homework (30–40 min), and semi-structured interviews (90–120 min)	Utilizing Bowen Family Systems Theory in supervision can address challenges like unawareness, heightened anxiety, feelings of isolation, restricted freedom, and insufficient knowledge about IP. The multimodal approach significantly improved self-awareness in MFT students, helping them distinguish their thoughts and emotions and separate emotional from intellectual processes.
Zanchetta et al., 2020	Research article	Experimental (randomized controlled trial) (quantitative)	103	Young employees in industrial sector Men (67%), women (33%) Mean age = 18.39 years (SD = 2)	*Quantitative* Impostor phenomenon Motivation Satisfaction Utility Career management Content-related knowledge Attributional style Goal attainment Self-efficacy Tendency to cover up errors Fear of negative evaluation	Dyadic coaching or training in a group setting with 8–10 participants [9 × 40 min units spread over three sessions (each 120 min) with 2-week intervals between the sessions] No intervention control group	Training intervention is superior in regard to convey content-related knowledge but shows more slightly results in reducing IP scores whereas dyadic coaching sessions are especially effective in reducing IP scores sustainably. Coaching improved self-enhancing attributions and self-efficacy and reduced the tendency to cover up errors as well as the fear of negative evaluation.

#### Study characteristics

Different research designs were used to guide each study. The main characteristics of the studies are reported in Table 3.

**TABLE 3:** Main study characteristics.

**Study design**	**Quantitative**	**Qualitative**	**Mixed-method**
	Danhauer et al., 2019; Metz et al., 2020; Zanchetta et al., 2020; Fainstad et al., 2022; Ogunyemi et al., 2022; Liu et al., 2023; Mann et al., 2023	Harte and McGlade, 2018; Popovic, 2020; DeCandia Vitoria, 2021; Magro, 2022	Haney et al., 2018; Gold et al., 2019; Baumann et al., 2020; Chang et al., 2022; Deshmukh et al., 2022
**Sample size**	***N* < 10**	**10 < *N* < 100**	***N* > 100**
	Harte and McGlade, 2018; DeCandia Vitoria, 2021; Magro, 2022	Danhauer et al., 2019; Gold et al., 2019; Baumann et al., 2020; Chang et al., 2022; Deshmukh et al., 2022	Haney et al., 2018; Metz et al., 2020; Zanchetta et al., 2020; Fainstad et al., 2022; Ogunyemi et al., 2022; Liu et al., 2023; Mann et al., 2023

The robustness of the study design varied widely from one study to another. Only a few studies involved a randomized controlled trial comparing the effects of their interventions with a control group (i.e., no intervention or a waiting list) (Zanchetta et al., 2020; Fainstad et al., 2022; Liu et al., 2023; Mann et al., 2023). Most other studies measured the variables before and after the intervention. Three studies used a cross-sectional design (Haney et al., 2018; Baumann et al., 2020; Deshmukh et al., 2022) and also collected qualitative data on the interventions (e.g., feedback). Finally, two studies used a descriptive (Magro, 2022) or interpretative (Popovic, 2020) phenomenological approach. Regarding sample size, there is considerable variability. For example, the study by Mann et al. (2023) includes more than a thousand participants while DeCandia Vitoria (2021) study presents a case study.

The research objectives formulated in each study also contributed to the heterogeneity between them. While several specifically addressed IP and evaluated the effects of the proposed intervention (e.g., Zanchetta et al., 2020), others situated their objectives within a broader framework (e.g., Fainstad et al., 2022; Mann et al., 2023). It should be noted that very few studies examined the IP issue in isolation: most evaluated several variables simultaneously.

#### Intervention characteristics

Although intervention models varied from one study to another, common characteristics make it possible to identify trends (Table 4).

**TABLE 4:** Main intervention characteristics.

**Intervention type**	**Counseling**	**Training**
	Coaching: Harte and McGlade, 2018; Zanchetta et al., 2020; Fainstad et al., 2022; Magro, 2022; Mann et al., 2023	Haney et al., 2018; Danhauer et al., 2019; Metz et al., 2020; Zanchetta et al., 2020; Chang et al., 2022; Deshmukh et al., 2022; Ogunyemi et al., 2022; Liu et al., 2023
	Clinical supervision: Popovic, 2020; DeCandia Vitoria, 2021	
	Peer and support groups: Gold et al., 2019; Baumann et al., 2020	
**Modality (setting)**	**Group**	**Individual**
	Haney et al., 2018; Danhauer et al., 2019; Gold et al., 2019; Baumann et al., 2020; Popovic, 2020; Zanchetta et al., 2020; Chang et al., 2022; Deshmukh et al., 2022; Fainstad et al., 2022; Ogunyemi et al., 2022; Mann et al., 2023 (for training)	Harte and McGlade, 2018; Metz et al., 2020; Zanchetta et al., 2020; DeCandia Vitoria, 2021; Magro, 2022; Liu et al., 2023 (for coaching)
**Duration**	**Single session**	**Multiple sessions**
	Haney et al., 2018; Chang et al., 2022; Deshmukh et al., 2022; Ogunyemi et al., 2022	Harte and McGlade, 2018; Danhauer et al., 2019; Gold et al., 2019; Baumann et al., 2020; Metz et al., 2020; Popovic, 2020; Zanchetta et al., 2020; DeCandia Vitoria, 2021; Fainstad et al., 2022; Magro, 2022; Liu et al., 2023; Mann et al., 2023
**Objectives**	**Addressing specifically IP**	**Broader program**
	Haney et al., 2018; Metz et al., 2020; Popovic, 2020; Zanchetta et al., 2020; DeCandia Vitoria, 2021; Chang et al., 2022; Deshmukh et al., 2022; Magro, 2022; Ogunyemi et al., 2022; Liu et al., 2023	Harte and McGlade, 2018; Danhauer et al., 2019; Gold et al., 2019; Baumann et al., 2020; Fainstad et al., 2022; Mann et al., 2023

First, two major types of approaches emerge among these models. More specifically, half the interventions were based on practices carried out in the context of counseling. These included coaching interventions, clinical supervision, and support groups. Additionally, almost all the other interventions adopted a professional training approach. Only one study (Zanchetta et al., 2020) compared two types of intervention (individual coaching and group training).

Several interventions based on principles of counseling aimed to foster openness and sharing of experiences among peers, in order to break the sense of isolation that characterizes IP. This is particularly true of the reflection and support groups studied by Gold et al. (2019) and the small group discussion sessions offered by Baumann et al. (2020). In the field of supervision, DeCandia Vitoria (2021) adopted an approach defined by simultaneous use of narrative therapy and interpersonal neurobiology. In her study, Popovic (2020) combined psychoeducation with group supervision and individual exercises. Finally, coaching interventions most often addressed the behavioral, cognitive, and emotional aspects of IP. These interventions are based on the coaching alliance, especially in the case of an individual approach (Zanchetta et al., 2020; Magro, 2022).

Training courses are essentially aimed at enhancing knowledge, interpersonal skills or know-how. Some of the selected studies involved purely technical skills related to the activity (e.g., *medical improvisational techniques*, Deshmukh et al., 2022), others more cross-disciplinary or relational skills (e.g., *adapting communication style, managing conflicts*, etc., Danhauer et al., 2019). The intervention proposed by Metz et al. (2020) consisted of an online training module on IP presenting more appropriate coping mechanisms. Some training sessions took the form of participative and reflective pedagogical workshops (e.g., Haney et al., 2018; Ogunyemi et al., 2022), with the goal of promoting feelings of liberation and empowerment.

Interventions can also be distinguished based on whether they involve group or individual modalities. While most of the selected studies trended toward group interventions, some were individual. Duration of the interventions, which was highly variable, is another characteristic differentiating the studies. Duration ranged from a single 1-h session (e.g., Deshmukh et al., 2022) to several months (e.g., Mann et al., 2023). Nevertheless, most interventions involved multiple sessions. Finally, some interventions were also part of a broader program, while others focused specifically on the IP.

#### Characteristics of identified effects

Although most studies concluded that the proposed intervention was effective in treating IP, these conclusions were based on evidence that varied from study to study. Indeed, the type of design and the nature of the data collected undeniably influenced the effects identified and conclusions. A distinction can be drawn between the main effects reported by studies that collected quantitative data, using an experimental or pre-experimental design (pre-intervention and post-intervention measures), and the effects identified by studies that reported qualitative data.

Among the studies that based their conclusions on quantitative data, some compared an experimental condition with a control condition. In a randomized controlled trial, they demonstrated the effectiveness of the proposed intervention compared to the absence of intervention. For example, after 6 months of professional coaching intervention, Fainstad et al. (2022) found a significant reduction in IP in female participants compared to those in the control group. The study by Zanchetta et al. (2020) compared two experimental conditions (training and coaching) and a control condition. It highlighted the lasting effectiveness of a coaching intervention in reducing IP issues. These authors concluded that training was less effective than coaching in reducing IP issues but was superior for acquiring associated knowledge. Other studies analyzed the variations in scores obtained by participants before and after the intervention, without comparison with a control group. For example, Metz et al. (2020) reported the beneficial effects of their online training module, as evidenced by a significant decrease in scores on the Clance Impostor Phenomenon Scale (CIPS) at the end of the semester, 4 months after their intervention.

Studies that reported qualitative data on the effects of interventions revealed complementary information. Some presented evaluations related to the satisfaction or experience of the participants, which were usually favorable or very favorable (e.g., Danhauer et al., 2019, Baumann et al., 2020; Deshmukh et al., 2022). This positive outcome led the authors to conclude that their intervention adequately addressed the issues. For example, participants in the intervention conducted and evaluated by Gold et al. (2019) reported that it contributed to improving their wellbeing, self-awareness, and empathy skills. Similarly, according to Haney et al. (2018), their training achieved its objectives, as the participants expressed feelings of liberation and empowerment after the workshop.

Finally, exploratory studies, whether descriptive phenomenology or case studies, clearly illustrate the mechanisms on which interventions rely to promote the reduction of IP, which they do not otherwise measure. Thus, Harte and McGlade (2018) concluded that coaching helped give direction and meaning to individual experience, which in turn helped counteract feelings of imposture. Magro (2022), pointed to the counseling and the tools and techniques learned, which enabled clients to improve their awareness and manage their feelings of imposture. Similarly, the clinical supervision proposed by DeCandia Vitoria (2021) to a therapist seemed to enable her to regain confidence, reduce her feelings of shame and fear, and strengthen her self-empathy.

### Studies carried out without a research design

Table 5 includes references presenting interventions carried out without a research design.

**TABLE 5:** References with no study design or that did not test an intervention.

References	Publication type	Population	Intervention type (theoretical framework)	Key ideas
Arleo et al., 2021	Editorial	Physicians	Recommendations for personal and institutional strategies (CBT)	The approach systematically consists of recognizing the IP to treat it using CBT techniques (including acknowledging and recognizing one’s thoughts and fears as distorted; using concrete evidence of achievements such as board certification and positive feedback from colleagues as proof of the distortion) and rely on peer support and mentorship.
Armstrong and Shulman, 2019	Commentary	Physicians	Personal strategies, mentoring and targeted programs (departmental, institutional, and professional)	Neurologists facing IP can implement conscious alterations in their thinking and behavior to counteract IP tendencies. It is essential for departments and mentors to proactively recognize faculty members grappling with IP and provide customized approaches for overcoming it, in addition to offering individualized career advancement opportunities.
Chassangre and Callahan, 2015a	Book chapter	General population	Individual psychotherapy techniques (CBT)	By combining a self-observation approach with cognitive restructuring methods and behavioral exercises, it is possible to break the behavioral and cognitive cycle specific to IS. Techniques such as behavioral journaling, assertiveness training and role-playing can help individuals with IS.
Chassangre and Callahan, 2015b	Book chapter	General population	Preventive interventions and personal strategies	Define “SMART” goals, identify obstacles, regulate the need for approval and perfectionist cognitions, share thoughts and representations with resource people, foster mentoring relationships.
Chassangre and Callahan, 2017	Literature review	General population	Therapeutic interventions and practices for individual therapy (mainly CBT), group therapy, discussion groups with general or specific themes about the IS	The issues at stake in the management of IP essentially involve identifying dysfunctional attitudes and learning more adapted cognitions and behaviors; constructing a more favorable attribution process; establishing a positive and realistic self-image; reducing of the person’s dependence on external evaluation.
Clance and Imes, 1978	Article	*High achieving women* (*N* = 150)	Group therapy (or interactional group) using several therapeutic approaches (CBT and Gestalt therapy)	Combination of such therapeutic interventions (supported by weekly homework assignments to practice new ideas about self-image) in conjunction with a commitment to change makes it possible to experience a decrease in feelings of imposture and associated behaviors.
Clance, 1985	Book chapter	General population	Individual and group psychotherapy techniques (CBT)	The main areas of therapy are defined, and CBT-inspired tools are used to achieve the therapeutic objectives: *Examining the mask and break the cycle* (self-observation exercises, behavioral exercises, and cognitive restructuring); *overcoming IP fears and guilts* (psycho-educational discourse, downward arrow technique); *learning to say no and enjoying success (diary of compliments, diary of attributions*).
Clance et al., 1995	Article	Women	Group therapy (feminist therapy)	Therapeutic group’s social and interpersonal setting has the potential to counterbalance and replace the initial messages received from family and society. The group can create a potent and efficient space for increasing self-awareness, encouraging observation, fostering exploration, and instigating transformative shifts in impostor-related emotions and behaviors.
Dowd and Davidhizar, 1997	Article	Supervisors (in the healthcare field)	Techniques to help supervisors, especially new supervisors, in developing a positive self-image	The approach consists of self-identifying the IP, the establishing a mentorship and collaborating with experienced supervisors, maintaining, developing and reinforcing professional skills and methods to promote a positive self-image.
Harvey and Katz, 1985	Book chapter	General population	Individual psychotherapy techniques (CBT)	The intervention addresses the cognitive, emotional and behavioral components of IP. After identifying and naming it (psychoeducation), the aim is to recognize the moments at which it is most likely to manifest, and through cognitive restructuring to adopt alternative thoughts. Relaxation techniques, emotional openness and shared experiences are combined to help change IP habits and revise the self-ideal.
Jaqua et al., 2021	Brief article	Physicians	Recommendations for personal strategies	Seven steps: “*Acknowledge feelings, Set reasonable expectations and goals, Find a mentor, Teach others, Question the status quo, Track successes to increase internal validation, Practice self-compassion.*” (p. 40)
Matthews and Clance, 1985	Article	Psycho-therapy clients	Group therapy or short-term growth-oriented group (CBT)	Group setting helps break down the sense of isolation associated with IP issues. Therapeutic intervention consists of labeling and developing awareness about IP feelings (reading suggestions). Examining the origins of the impostor phenomenon in clients is another major focus of the therapy. The rational emotive techniques or desensitization may be helpful for countering fear of failure.
Parkman and Beard, 2008	Brief article	Employees in the higher education field	Human resources managerial strategies to address impostor behaviors on succession planning in higher education	Two complementary intervention components: one at an individual level (feedback, human resource programs: peer group, mentorship and discussions addressing work situations) and the other at an organizational level (fostering awareness, considering what types of messages are being sent via organizational processes and policies).
Ramsey and Spencer, 2019	Brief article	Medical interns	Educational session (psychoeducation about IP, small group discussions, role-play exercise)	Interns practiced addressing IS thought patterns and adjusting their responses with the help of facilitator feedback, role-playing, and targeted practice. Following the session, there was a marked rise in their willingness to recognize their areas of uncertainty, seek help, and feel comforted by the understanding that they are not alone in their fears.
Steinberg, 1987	Article	Women (*N* = 7–9 per group)	Group therapy (known as *The Impostor Group*): discussion in small group and sharing among peers	One significant advantage of the group setting is the invaluable knowledge exchange among women. The IP is presented as not merely an individual psychological issue; rather, it is a widespread problem that requires attention from individuals involved in the upbringing and societal integration of girls and women.

IP, impostor phenomenon; IS, impostor syndrome; CBT, cognitive behavioral therapy.

#### Study characteristics

Most of the references classified in this section are articles published in peer-reviewed journals and describe various intervention models carried out in diverse contexts (Clance and Imes, 1978; Matthews and Clance, 1985; Steinberg, 1987; Clance et al., 1995; Parkman and Beard, 2008; Ramsey and Spencer, 2019). These articles present a description of the IP, the intervention carried out and its repercussions, as well as recommendations for practitioners wishing to develop a similar approach. However, the interventions described were neither carried out nor evaluated using a research design. Most often, neither the IP nor other variables were measured. These articles therefore present a professional view of IP interventions, outside the field of empirically based interventions. Therefore, the results reported cannot be compared to those obtained by the studies reported in the previous section. However, it is important to present them in order to better understand the current state of knowledge regarding IP interventions.

Among the selected references, one literature review (Chassangre and Callahan, 2017) and one commentary (Armstrong and Shulman, 2019) address general aspects and list guidelines as well as recommendations for interventions addressing IP. Three others provide scientifically supported recommendations, but their implementation is not described (Dowd and Davidhizar, 1997; Arleo et al., 2021; Jaqua et al., 2021). In addition, this section includes a chapter from Clance (1985) and one from Harvey and Katz (1985), pioneering authors on the subject of IP. These authors present interventions and recommendations based on their clinical experience. Similarly, the two chapters from the work of Chassangre and Callahan (2015a,b) synthesize the contributions of the previous authors and integrate them into their proposed intervention on IP, within the psychotherapeutic framework.

#### Intervention characteristics

In the references identified, two main types of interventions can be distinguished, those focusing on psychotherapy and those making recommendations for personal strategies.

Most of the interventions described fall within the field of psychotherapy and include both group therapy (Clance and Imes, 1978; Matthews and Clance, 1985; Steinberg, 1987; Clance et al., 1995) and individual interventions (Harvey and Katz, 1985; Dowd and Davidhizar, 1997; Chassangre and Callahan, 2015a,b). Some authors also make recommendations for both modes of intervention (Clance, 1985; Chassangre and Callahan, 2017). In terms of theoretical framework, several references adopt an approach derived from cognitive behavioral therapy (CBT) (Clance, 1985; Harvey and Katz, 1985; Matthews and Clance, 1985; Chassangre and Callahan, 2015a,b, 2017). For example, Clance and Imes (1978) proposed an intervention integrating the contributions of CBT and Gestalt therapy. A few years later, Clance et al. (1995) suggested a feminist-oriented therapeutic group. An intervention put forward by Ramsey and Spencer (2019) resembles training, but also integrates some aspects of counseling, combining a psychoeducational component focusing on IP with small-group discussions and role-playing exercises.

Articles recommending interventions based on personal strategies to reduce IP also address the cognitive, emotional and behavioral components of the phenomenon (e.g., Chassangre and Callahan, 2015a; Arleo et al., 2021; Jaqua et al., 2021). Among these recommendations, it is interesting to note that the approaches described have much in common with each other, but also with coaching (e.g., Zanchetta et al., 2020). For example, Jaqua et al. (2021) describe a seven-step counseling approach that involves acknowledging one’s own feelings and practicing self-compassion, yet also recommend finding a mentor in order to be able to rely on external resources. Overall, these interventions are more briefly described than those based on psychotherapy.

#### Characteristics of identified effects

Most studies conducted without a research design offer a relatively brief description of the impact of interventions on participants. The effects described are exclusively qualitative in nature, or are estimates of expected effects, according to the clinical or professional experience of the authors. Nevertheless, the information is sufficiently complete to enable several observations.

Firstly, authors who have opted for a group intervention approach argue that it can help break the feeling of isolation often experienced with IP (e.g., Matthews and Clance, 1985). In this regard, Clance et al. (1995) suggest that their feminist-oriented therapeutic intervention makes it possible to counteract and substitute the familial and social influences initially received. Steinberg (1987) describes a peer discussion group (*The Impostor Group*) and identifies the sharing of knowledge and experiences as one of the main strengths of the intervention. However, individual support may also be indicated in certain cases, as described by Matthews and Clance (1985), offering an opportunity to personalize the treatment of IP. Finally, some researchers suggest that training-based interventions enable the transmission of IP-related knowledge and its subsequent reevaluation on a personal level. For example, Ramsey and Spencer (2019) emphasize that their intervention enables participants to recognize their knowledge gaps more serenely and to seek help without concealing their feelings of imposture.

## Discussion

The aim of this scoping review, which situates the contribution of 31 references, is to address three main questions arising from the literature on interventions for IP. The first concerns the types of studies and methodologies that currently exist regarding such interventions. The second focuses on the modalities and characteristics of these interventions, and the third concerns their effects. These three questions are discussed below, followed by the limitations of this review and future perspectives for this field of research.

### Type of studies and methodologies

Most of the references included in this scoping review are articles in peer-reviewed journals. However, not all present a scientific investigative approach. Those supported by a scientific research design are from after 2018, and used various methodologies. A majority proposed a quantitative design (e.g., Danhauer et al., 2019), while others relied on mixed or purely qualitative data (e.g., Haney et al., 2018). Research design robustness varied across studies: some presented randomized controlled trials (e.g., Fainstad et al., 2022) while others adopted exploratory approaches (e.g., Magro, 2022). Sample sizes also varied, ranging from individual case (e.g., DeCandia Vitoria, 2021) to studies including over a thousand participants (e.g., Mann et al., 2023).

That said, many of the interventions cited were conducted without the aid of a scientific research design. For example, some articles described professional experiments (e.g., Clance and Imes, 1978). Such interventions cannot be considered as evidence-based practices and are more exploratory in nature. However, the methodological recommendations for scoping reviews, unlike systematic reviews, make it possible to include these publications. In addition to the recent developments identified in this scoping review, this may also explain some of the differences found with the most recent published systematic reviews on IP (Bravata et al., 2020; Gullifor et al., 2023). Thus, it is appropriate to be cautious when extracting the best practices from this literature to guide the development of future interventions.

### Modalities and characteristics of interventions

Before discussing the intervention characteristics, it is important to note that most authors agreed on a common definition of IP, one that is consistent with its original conceptualization (e.g., Clance and Imes, 1978; Clance, 1985). Indeed, the definition of IP is not discussed in most of the studies selected, despite the persistent lack of conceptual clarity relating to IP. This observation echoes that of Gullifor et al. (2023) when challenging “*the implicit assumption that the conceptualization of IP is thoroughly and soundly developed*” (p. 2).

In fact, according to the authors, IP manifests on several levels: cognitive, emotional, and behavioral. Many interventions, whether psychotherapeutic or non-psychotherapeutic, take a CBT-inspired approach to address IP (e.g., Clance, 1985; Chassangre and Callahan, 2017; Gold et al., 2019). In particular, Chassangre and Callahan’s (2015a) interventions draw on intervention techniques described by Clance (1985) (e.g., *Attribution Diary, Downward Arrow Technique*, etc.), to influence the dynamics of the *impostor cycle*. Baumann et al. (2020) also point to the influence of CBT in their approach to IP discussion groups. However, interventions based on other conceptual frameworks, such as positive psychology (Fainstad et al., 2022; Mann et al., 2023), narrative therapy and interpersonal biology (DeCandia Vitoria, 2021) or Gestalt (Clance and Imes, 1978), offer another perspective on the beliefs underlying IP (e.g., representations of success, professional skills, beliefs about one’s own abilities, etc.). They also suggest behavioral coping strategies, such as techniques to prevent procrastination (e.g., Metz et al., 2020).

Furthermore, pioneering authors (Clance and Imes, 1978; Clance, 1985; Clance et al., 1995) considered IP to be a psycho-social and interpersonal problem. They emphasized the influence of social norms and stereotypes on the emergence of feelings of imposture, particularly among women. For this reason, Clance et al. (1995) proposed a group therapy approach aimed at overcoming the intrapsychic and sociocultural determinants that can lead to the development and maintenance of impostor feelings. IP continues to be viewed as partly a social and collective phenomenon rather than a strictly individual issue in some recent research (e.g., Haney et al., 2018; Baumann et al., 2020; Popovic, 2020; Arleo et al., 2021). Many interventions thus account for the role played by the social dimension of the work environment (e.g., Danhauer et al., 2019; Gold et al., 2019; Deshmukh et al., 2022) and aim to break the sense of isolation associated with IP. Although some authors opt for an individual approach (e.g., Harte and McGlade, 2018; Metz et al., 2020; DeCandia Vitoria, 2021); according to Steinberg (1987), Parkman and Beard (2008), and Armstrong and Shulman (2019), IP is systemic in nature and therefore needs to be addressed at the organizational or social level. It may therefore be relevant to address it within educational programs, integration strategies, and human resource management policies. This is consistent with recent theoretical developments on IP at work, which suggests that it may emerge from the social context (e.g., Kark et al., 2022).

Finally, it should be noted that two main intervention modalities appear: those based on discussion and counseling, and those focused on training. Most approaches incorporate a psycho-educational component, either provided by the facilitator (e.g., Haney et al., 2018; Ogunyemi et al., 2022), or through suggested readings (Matthews and Clance, 1985; Steinberg, 1987). Role-playing is a technique frequently mentioned in this context and can encourage the expression of feelings as well as regulation of IP manifestations (e.g., Ramsey and Spencer, 2019; Deshmukh et al., 2022).

Following these observations, intervention methods such as training or counseling seem to be the most popular, as are group-based interventions. The transmission of IP-related knowledge emerges as a trend to be retained as a good practice for interventions. Futures research should also clarify the conceptualization they adopt regarding IP.

### Effects of interventions

As mentioned above, the impact of the interventions described differs according to the type of intervention and design used. In this section, the effects reported by interventions based on counseling and training, as well as the effects of more specific intervention modalities (e.g., role-playing), are discussed separately.

Among interventions based on counseling, coaching is repeatedly presented as being effective, as are peer exchange and support groups (e.g., Harte and McGlade, 2018; Baumann et al., 2020; Mann et al., 2023). These interventions seem to promote openness and awareness. They also encourage effort at the level of mentalization, as much as the concrete implementation of coping strategies (e.g., Harte and McGlade, 2018; Magro, 2022). Furthermore, in a group context, the impact of the facilitator’s counseling with participants seems to be amplified by the relationships among the participants themselves (e.g., Steinberg, 1987; Baumann et al., 2020; Fainstad et al., 2022). More specifically, this type of intervention can foster connection and a sense of social belonging (Gold et al., 2019).

Training-based interventions are relevant for knowledge transmission. Several studies have shown a post-intervention increase in IP-related knowledge (e.g., Deshmukh et al., 2022; Ogunyemi et al., 2022). In addition, training can promote a decrease in IP (e.g., Metz et al., 2020), as well as positive feelings of liberation, empowerment, and connection with the peer group (e.g., Haney et al., 2018; Gold et al., 2019).

That said, when comparing coaching and training, the study by Zanchetta et al. (2020) is particularly interesting. According to these authors, coaching operates on multiple levels and produces a significant and lasting reduction in IP issues. However, training remains a more favorable approach for acquiring knowledge associated with IP. In the absence of studies comparing the different approaches, it is difficult to definitively establish the superiority of individual coaching over other modalities of intervention (i.e., training, support groups, etc.).

Future research could build on the promising approaches that counseling interventions seem to offer, by incorporating a psychoeducational component. Additionally, it appears that the group intervention modality seems appropriate for breaking the isolation related to IP. Although the current state of the literature provides interesting insights into the effectiveness of interventions, but it still needs to be enriched. It appears that mixed-method study designs, incorporating both quantitative measures and the analysis of qualitative data, would allow for an understanding of both if and how the interventions are useful and effective.

### Limitations and futures research directions

This study has certain limitations that should be mentioned. A first limitation stems from the nature of the references included. Indeed, almost half do not present any research design. Consequently, the generalizability of their findings is extremely limited. Moreover, many studies that use a research design are exploratory and very few adopt a true experimental design. Most often, approaches to assessing the effects of intervention on IP are insufficiently robust. For example, some studies do not measure IP directly (e.g., Danhauer et al., 2019), others have no post-intervention measurement (e.g., Deshmukh et al., 2022), or assess intervention quality exclusively via self-reported satisfaction (e.g., Baumann et al., 2020). It is therefore difficult to generalize findings about the effects of these interventions on IP.

A second limitation arises from incomplete information regarding the characteristics and modalities of the interventions. Indeed, several studies offer a limited description of the design and implementation of the intervention for the IP. Aspects such as an overview of intervention content, duration, context, intervention mode and nature of interactions are not systematically reported by the authors (e.g., Clance and Imes, 1978; Baumann et al., 2020; DeCandia Vitoria, 2021). Details about the characteristics of these interventions are often lacking, even when they are essential.

A third limitation concerns the heterogeneity of the references collected and the interventions described, which constitutes an obstacle to comparing these interventions. For example, it would be inappropriate to compare the findings of an exploratory study (Harte and McGlade, 2018) with those of a randomized controlled trial (Mann et al., 2023). Similarly, it would not be reasonable to compare an intervention carried out over a period of 1 h (Deshmukh et al., 2022) with one carried out over a period of 1 day (Haney et al., 2018) or several months (Zanchetta et al., 2020). Thus, while most interventions on IP have recognizable positive effects, it remains difficult to establish precise criteria when it comes to evaluating the effectiveness of interventions on IP.

A fourth limitation regards the student population samples used in many studies included in this scoping review. Although we have restricted the eligible population to students in internship situations or already working in a professional environment, it is legitimate to wonder whether this “in-between” situation raises the same issues as those encountered in a strictly professional environment. In order to truly understand and address the organizational issues associated with IP, it would be important for future research to investigate a professional audience, at the very heart of the activity.

While this scoping review cannot pinpoint the *ideal intervention* for all the reasons mentioned above, it nonetheless identifies certain favorable trends for effectively intervening on IP. For example, bidirectional approaches, which promote exchange and openness, have particularly favorable impact on IP. Moreover, it is essential to consider different modes of IP expression and psychoeducation when developing an intervention. That said, it would also be relevant to promote reflective work on the causes of IP (psycho-social, educational, systemic origins, etc.), as well as the role of the social environment in reinforcing the beliefs and patterns associated with it. Finally, this scoping review highlights the fact that group support (as opposed to individual intervention) appears advantageous. However, customizing the intervention should be considered, to allow participants to develop a better sense of self. Developing individuals’ psychological resources by promoting self-knowledge also seems a particularly relevant research focus. According to Gullifor et al. (2023), the impostor phenomenon may emerge from an incongruence between one’s own self-concept and other self-concept. Therefore, any research perspective aimed at developing a more accurate vision of oneself is interesting. These avenues could be explored not only in future research, but also by those working with people reporting IP in an organizational context.

In summary, future research should respond to a dual contextual challenge: a gap to be bridged between the small number of scientific studies and the abundant lay literature on IP; and a lack of conceptual clarity regarding IP. From this perspective, future research should use more rigorous methodology in terms of variable measurement (e.g., systematic pre- and post-intervention measurements of the variables studied) and ensure that robust research designs are used (e.g., experimental or quasi-experimental designs). It may also be relevant to investigate the psychological processes involved in the development of IP in order to intervene on them. Regarding the framework and design of interventions, future studies could integrate a psychosocial understanding of the phenomenon into the aspects described above. This would distinguish them from the lay literature, build on advice and recommendations associated with purely individual strategies.

## Conclusion

This scoping review is aimed at researchers and professionals interested in interventions for IP and presents the current state of knowledge in this regard. It discusses the different types of studies and methodologies emerging from this field of research, the modalities and characteristics of interventions, and their effects. It also provides insight into gaps and perspectives for future practices and research.

In conclusion, the study of interventions addressing IP is an emerging field of research that is recently experiencing considerable progress and still requires further exploration. Indeed, while the *imposter syndrome* is a topic that seems to have gained in popularity and is a common experience, there could be a risk involved in trivializing it. It represents a psychological health issue at work, ultimately affecting the proper functioning of organizations.

Although conceptual issues remain to be resolved to better understand IP, it is just as urgent to develop interventions aimed at reducing it, in view of its implications for people’s health and its negative consequences in the workplace. In light of the results reported in this scoping review, recognizing and educating individuals experiencing IP about its various manifestations, as well as offering support in a group context, appear to be important intervention strategies. Future interventions should aim to explore the psycho-social and educational influences, as well as the impact of the work environment on beliefs related to IP.

## Data availability statement

The original contributions presented in this study are included in this article/Supplementary material, further inquiries can be directed to the corresponding author.

## Author contributions

EP: Conceptualization, Data curation, Formal analysis, Funding acquisition, Investigation, Methodology, Project administration, Resources, Validation, Writing – original draft, Writing – review & editing. PD: Methodology, Project administration, Resources, Supervision, Validation, Visualization, Funding acquisition, Writing – original draft, Writing – review & editing. PM: Methodology, Project administration, Resources, Supervision, Validation, Visualization, Writing – original draft, Writing – review & editing. CM-K: Methodology, Project administration, Resources, Supervision, Validation, Visualization, Funding acquisition, Writing – original draft, Writing – review & editing.

## References

[B1] Alrayyes S. Dar U. F. Alrayes M. Alghutayghit A. Alrayyes N. (2020). Burnout and imposter syndrome among Saudi young adults. the strings in the puppet show of psychological morbidity. *Saudi Med. J.* 41 189–194. 10.15537/smj.2020.2.24841 32020154 PMC7841628

[B2] Ares T. L. (2018). Role transition after clinical nurse specialist education. *Clin. Nurse Specialist* 32 71–80. 10.1097/NUR.0000000000000357 29419579

[B3] Arleo E. K. Wagner-Schulman M. McGinty G. Salazar G. Mayr N. A. (2021). Tackling impostor syndrome: a multidisciplinary approach. *Clin. Imaging* 74 170–172. 10.1016/j.clinimag.2020.12.035 33478806

[B4] Armstrong M. J. Shulman L. M. (2019). Tackling the imposter phenomenon to advance women in neurology. *Neurol. Clin. Pract.* 9 155–159. 10.1212/CPJ.0000000000000607 31041131 PMC6461421

[B5] Baumann N. Faulk C. Vanderlan J. Chen J. Bhayani R. K. (2020). Small-group discussion sessions on imposter syndrome. *MedEdPORTAL* 16:11004. 10.15766/mep_2374-8265.11004 33204832 PMC7666839

[B6] Bechtoldt M. (2015). Wanted: self-doubting employees—managers scoring positively on impostorism favor insecure employees in task delegation. *Pers. Individ. Dif.* 86 482–486. 10.1016/j.paid.2015.07.002

[B7] Bernard N. S. Dollinger S. J. Ramaniah N. V. (2002). Applying the big five personality factors to the impostor phenomenon. *J. Pers. Assess.* 78 221–233. 10.1207/S15327752JPA7802_07 12067196

[B8] Blondeau L. A. Awad G. H. (2018). The relation of the impostor phenomenon to future intentions of mathematics-related school and work. *J Career Dev.* 45 253–267. 10.1177/0894845316680769

[B9] Brauer K. Wolf A. (2016). Validation of the german-language clance impostor phenomenon scale (GCIPS). *Pers. Individ. Dif.* 102 153–158.

[B10] Bravata D. M. Watts S. A. Keefer A. L. Madhusudhan D. K. Taylor K. T. Clark D. M. (2020). Prevalence, predictors, and treatment of impostor syndrome: a systematic review. *J. Gen. Intern. Med.* 35 1252–1275. 31848865 10.1007/s11606-019-05364-1 PMC7174434

[B11] Byrnes K. D. Lester D. (1995). The imposter phenomenon in teachers and accountants. *Psychol. Rep.* 77:350. 10.2466/pr0.1995.77.1.350 7501775

[B12] Chang S. Lee H. Y. Anderson C. Lewis K. Chakraverty D. Yates M. (2022). Intervening on impostor phenomenon: prospective evaluation of a workshop for health science students using a mixed-method design. *BMC Med. Educ.* 22:802. 10.1186/s12909-022-03824-7 PMC9673315 36397022

[B13] Chassangre K. (2016). *La Modestie Pathologique : Pour Une Meilleure Compréhension du Syndrome de L’imposteur.* Thèse doctorale. Toulouse: Université Toulouse le Mirail.

[B14] Chassangre K. Callahan S. (2015a). “Outils de prise en charge cognitive et comportementale du syndrome,” in *Traiter la dépréciation de soi. Le syndrome de l’imposteur*, ed. Dunod (Paris: Dunod), 135–169.

[B15] Chassangre K. Callahan S. (2015b). “Exercices et méthodes pour consolider la prise en charge,” in *Traiter la dépréciation de soi. Le syndrome de l’imposteur*, ed. Dunod (Paris: Dunod), 170–180.

[B16] Chassangre K. Callahan S. (2017). J’ai réussi, j’ai de la chanc je serai démasqué: revue de littérature du syndrome de l’imposteur. *Pratiques Psychologiques* 23 97–110. 10.1016/j.prps.2017.01.001

[B17] Chrisman S. M. Pieper W. A. Clance P. R. Holland C. L. Glickauf-Hughes C. (1995). Validation of the clance imposter phenomenon scale. *J. Pers. Assess.* 65 456–467. 10.1207/s15327752jpa6503_6 16367709

[B18] Clance P. R. (1985). “Part three: Taking off the mask,” in *The impostor phenomenon: Overcoming the fear that haunts your success*, ed. Peachtree Pub Ltd (Atlanta: Peachtree Pub Ltd), 129–191.

[B19] Clance P. R. Dingman D. Reviere S. L. Stober D. R. (1995). Impostor phenomenon in an interpersonal/social context. *Women Therapy* 16 79–96. 10.1300/J015v16n04_07

[B20] Clance P. R. Imes S. A. (1978). The imposter phenomenon in high achieving women: dynamics and therapeutic intervention. *Psychother. Theory Res. Pract.* 15 241–247. 10.1037/h0086006

[B21] Clance P. R. O’Toole M. A. (1987). The imposter phenomenon: an internal barrier to empowerment and achievement. *Women Therapy* 6 51–64. 10.1300/J015V06N03_05

[B22] Colquhoun H. L. Levac D. O’Brien K. K. Straus S. Tricco A. C. Perrier L. (2014). Scoping reviews: time for clarity in definition, methods, and reporting. *J. Clin. Epidemiol.* 67 1291–1294. 10.1016/j.jclinepi.2014.03.013 25034198

[B23] Crawford W. S. Shanine K. K. Whitman M. V. Kacmar K. M. (2016). Examining the impostor phenomenon and work-family conflict. *J. Manag. Psychol.* 31 375–390. 10.1108/JMP-12-2013-0409

[B24] Danhauer S. C. Tooze J. A. Barrett N. A. Blalock J. S. Shively C. A. Voytko M. L. (2019). Development of an innovative career development program for early-career women faculty. *Glob. Adv. Health Med.* 8:2164956119862986. 10.1177/2164956119862986 31360616 PMC6636414

[B25] DeCandia Vitoria A. (2021). Experiential supervision: healing imposter phenomenon from the inside out. *Clin. Supervisor* 40 200–217.

[B26] Deshmukh S. Shmelev K. Vassilades L. Kurumety S. Agarwal G. Horowitz J. M. (2022). Imposter phenomenon in radiology: incidence, intervention, and impact on wellness. *Clin. Imaging* 82 94–99. 10.1016/j.clinimag.2021.11.009 34801842

[B27] Dowd S. B. Davidhizar R. (1997). Do you feel like an impostor? *Health Care Supervisor* 15 51–56. 10165429

[B28] Fainstad T. Mann A. Suresh K. Shah P. Dieujuste N. Thurmon K. (2022). Effect of a novel online group-coaching program to reduce burnout in female resident physicians: a randomized clinical trial. *JAMA Netw. Open* 5 1–14. 10.1001/jamanetworkopen.2022.10752 PMC9077483 35522281

[B29] Fried-Buchalter S. (1997). Fear of success, fear of failure, and the imposter phenomenon among male and female marketing managers. *Sex Roles* 37 847–859. 10.1007/BF02936343

[B30] Fruhan G. A. (2002). *Understanding Feelings of Fraudulence in the Early Professional Lives of Women.* Ann Arbor, MI: ProQuest Information & Learning.

[B31] Fujie R. (2010). Development of the state impostor phenomenon scale. *Jap. Psychol. Res.* 52 1–11. 10.1111/j.1468-5884.2009.00417.x

[B32] Gold J. A. Bentzley J. P. Franciscus A. M. Forte C. De Golia S. G. (2019). An intervention in social connection: medical student reflection groups. *Acad. Psychiatry* 43 375–380. 10.1007/s40596-019-01058-2 30963416

[B33] Gullifor D. P. Gardner W. L. Karam E. P. Noghani F. Cogliser C. C. (2023). The impostor phenomenon at work: a systematic evidence-based review, conceptual development, and agenda for future research. *J. Organ. Behav*. 45 234–251. 10.1002/job.2733

[B34] Haney T. S. Birkholz L. Rutledge C. (2018). A workshop for addressing the impact of the imposter syndrome on clinical nurse specialists. *Clin. Nurse Specialist* 32 189–194. 10.1097/nur.0000000000000386 29878930

[B35] Harte S. McGlade K. (2018). Developing excellent leaders - the role of executive coaching for GP specialty trainees. *Educ. Primary Care* 29 286–292. 10.1080/14739879.2018.1501770 30129393

[B36] Harvey J. C. (1981). *The impostor phenomenon and achievement: A failure to internalize success*. Temple University.

[B37] Harvey J. C. Katz C. (1985). “Chapter seven: Throwing away the mask,” in *If I’m so successful why do i feel like a fake: The impostor phenomenon*., ed. St Martin’s Press (New York, NY: St Martin’s Press), 205–240.

[B38] Jaqua E. E. Nguyen V. Park S. Hanna M. (2021). Coping with impostor syndrome. *Fam. Pract. Manag.* 28:40. 33973753

[B39] Kananifar N. Seghatoleslam T. Atashpour S. Hoseini M. Habil M. Danaee M. (2015). The relationships between imposter phenomenon and mental health in isfahan universities students. *Int. Med. J.* 22 144–146.

[B40] Kark R. Meister A. Peters K. (2022). Now you see me, now you don’t: a conceptual model of the antecedents and consequences of leader impostorism. *J. Manag.* 48 1948–1979. 10.1177/01492063211020358

[B41] Kets de Vries M. (2005). The dangers of feeling like a fake. *Harv. Bus. Rev.* 83 108–116. 16171215

[B42] LaDonna K. A. Ginsburg S. Watling C. (2018). “Rising to the level of your incompetence”: what physicians’ self-assessment of their performance reveals about the imposter syndrome in medicine. *Acad. Med.* 93 763–768. 29116983 10.1097/ACM.0000000000002046

[B43] Leach P. K. Nygaard R. M. Chipman J. G. Brunsvold M. E. Marek A. P. (2019). Impostor phenomenon and burnout in general surgeons and general surgery residents. *J. Surgical Educ.* 76 99–106. 10.1016/j.jsurg.2018.06.025 30122638

[B44] Leary M. R. Patton K. M. Orlando A. E.Wagoner Funk W. (2000). The impostor phenomenon: Self?perceptions, reflected appraisals, and interpersonal strategies. *J. Pers*. 68, 725–756. 10934688 10.1111/1467-6494.00114

[B45] Legassie J. Zibrowski E. M. Goldszmidt M. A. (2008). Measuring resident well-being: impostorism and burnout syndrome in residency. *J. Gen. Intern. Med.* 23 1090–1094. 10.1007/s11606-008-0536-x 18612750 PMC2517942

[B46] Leonhardt M. Bechtoldt M. N. Rohrmann S. (2017). All impostors aren’t alike–differentiating the impostor phenomenon. *Front. Psychol.* 8:1505. 10.3389/fpsyg.2017.01505 28936188 PMC5594221

[B47] Liu S. Wei M. Russell D. (2023). Effects of a brief self-compassion intervention for college students with impostor phenomenon. *J. Couns. Psychol.* 70 711–724. 10.1037/cou0000703 37498705

[B48] Lockwood C. Tricco A. C. (2020). Preparing scoping reviews for publication using methodological guides and reporting standards. *Nurs. Health Sci.* 22 1–4. 10.1111/nhs.12673 32115893

[B49] Magro C. (2022). From hiding to sharing. a descriptive phenomenological study on the experience of being coached for impostor syndrome. *Int. J. Evidence Based Coaching Mentoring* S16, 68–80. 10.24384/0409-b325

[B50] Mak K. K. L. Kleitman S. Abbott M. J. (2019). Impostor phenomenon measurement scales: a systematic review. *Front. Psychol.* 10:671. 10.3389/fpsyg.2019.00671 31024375 PMC6463809

[B51] Mann A. Shah A. N. Thibodeau P. S. Dyrbye L. Syed A. Woodward M. A. (2023). Online well-being group coaching program for women physician trainees: a randomized clinical trial. *JAMA Netw. Open* 6:e2335541. 10.1001/jamanetworkopen.2023.35541 37792378 PMC10551770

[B52] Matthews G. Clance P. R. (1985). Treatment of the impostor phenomenon in psychotherapy clients. *Psychother. Private Pract.* 3 71–81. 10.1300/J294v03n01_09

[B53] McElwee R. O. B. Yurak T. J. (2007). Feeling versus acting like an impostor: Real feelings of fraudulence or self-presentation? *Individ. Differ. Res*. 5.

[B54] McElwee R. O. B. Yurak T. J. (2010). The phenomenology of the impostor phenomenon. *Individ. Dif. Res.* 8 184–197. 37188407

[B55] Metz C. J. Ballard E. Metz M. J. (2020). The stress of success: an online module to help first-year dental students cope with the impostor Phenomenon. *J. Dental Educ.* 84 1016–1024. 10.1002/jdd.12181 32436247

[B56] Mir I. Kamal A. (2018). Role of workaholism and self-concept in predicting impostor feelings among employees. *Pak. J. Psychol. Res.* 33 413–427.

[B57] Munn Z. Peters M. D. Stern C. Tufanaru C. McArthur A. Aromataris E. (2018). Systematic review or scoping review? Guidance for authors when choosing between a systematic or scoping review approach. *BMC Med. Res. Methodol*. 18, 1–7. 30453902 10.1186/s12874-018-0611-x PMC6245623

[B58] Neureiter M. Traut-Mattausch E. (2016). An inner barrier to career development: preconditions of the impostor phenomenon and consequences for career development. *Front. Psychol.* 7:48. 10.3389/fpsyg.2016.00048 26869957 PMC4740363

[B59] Neureiter M. Traut-Mattausch E. (2017). Two sides of the career resources coin: career adaptability resources and the impostor phenomenon. *J. Vocat. Behav.* 98 56–69. 10.1016/j.jvb.2016.10.002

[B60] Ogunyemi D. Lee T. Ma M. Osuma A. Eghbali M. Bouri N. (2022). Improving wellness: defeating Impostor syndrome in medical education using an interactive reflective workshop. *PLoS One* 17:e0272496. 10.1371/journal.pone.0272496 35925925 PMC9352101

[B61] Oriel K. Plane M. B. Mundt M. (2004). Family medicine residents and the impostor phenomenon. *Fam. Med.* 36 248–252. 15057614

[B62] Parkman A. Beard R. (2008). Succession planning and the imposter phenomenon in higher education. *CUPA-HR J.* 59 29–36.

[B63] Peters M. D. J. Godfrey C. McInerney P. Munn Z. Tricco A. C. Khalil H. (2020). *Methodology for JBI Scoping Reviews.* Adelaide: The Joanna Briggs Institute.

[B64] Peters M. D. J. Godfrey C. M. Khalil H. McInerney P. Parker D. Soares C. B. (2015). Guidance for conducting systematic scoping reviews. *Int. J. Evid. Based Healthc.* 13 141–146. 26134548 10.1097/XEB.0000000000000050

[B65] Popovic M. (2020). *The Impact of Bowen Family Systems Theory Group Supervision Intervention with Master Level Marriage and Family Therapy Students Who Self-Identify with Having Imposter Phenomenon.* Doctoral dissertation. Texas: Texas Wesleyan University.

[B66] Ramsey J. L. Spencer A. L. (2019). Interns and imposter syndrome: proactively addressing resilience. *Med. Educ.* 53 504–505. 10.1111/medu.13852 30924160

[B67] Rohrmann S. Bechtoldt M. N. Leonhardt M. (2016). Validation of the impostor phenomenon among managers. *Front. Psychol.* 7:821. 10.3389/fpsyg.2016.00821 27313554 PMC4890534

[B68] September A. N. McCarrey M. Baranowsky A. Parent C. Schindler D. (2001). The relation between well-being, impostor feelings, and gender role orientation among canadian university students. *J. Soc. Psychol.* 141 218–232. 10.1080/00224540109600548 11372567

[B69] Sonnak C. Towell T. (2001). The impostor phenomenon in British university students: relationships between self-esteem, mental health, parental rearing style and socioeconomic status. *Pers. Individ. Dif.* 31 863–874. 10.1016/S0191-8869(00)00184-7

[B70] Steinberg J. A. (1987). Clinical interventions with women experiencing the impostor phenomenon. *Women Therapy* 5 19–26. 10.1300/J015V05N04_04

[B71] Tewfik B. A. (2022). The impostor phenomenon revisited: examining the relationship between workplace impostor thoughts and interpersonal effectiveness at work. *Acad. Manag. J.* 65 988–1018. 10.5465/amj.2020.1627

[B72] Topping M. H. (1983). *The Impostor Phenomenon: a Study of its Construct and Incidence in University Faculty Members.* Ann Arbor, MI: ProQuest Information & Learning.

[B73] Tricco A. C. Khalil H. Holly C. Feyissa G. Godfrey C. Evans C. (2022). Rapid reviews and the methodological rigor of evidence synthesis: a JBI position statement. *JBI Evid. Synth.* 20 944–949. 10.11124/JBIES-21-00371 35124684

[B74] Tricco A. C. Lillie E. Zarin W. O’Brien K. K. Colquhoun H. Levac D. (2018). PRISMA extension for scoping reviews (PRISMA-ScR): checklist and explanation. *Ann. Internal Med.* 169 467–473. 10.7326/M18-0850 30178033

[B75] Vergauwe J. Wille B. Feys M. De Fruyt F. Anseel F. (2015). Fear of being exposed: the trait-relatedness of the impostor phenomenon and its relevance in the work context. *J. Bus. Psychol.* 30 565–581. 10.1007/s10869-014-9382-5

[B76] Zanchetta M. Junker S. Wolf A.-M. Traut-Mattausch E. (2020). “Overcoming the fear that haunts your success” - the effectiveness of interventions for reducing the impostor phenomenon. *Front. Psychol.* 11:405. 10.3389/fpsyg.2020.00405 32499733 PMC7242655

