# Supplementary material for: Interventions addressing the impostor phenomenon: a scoping review
Source: Front Psychol. 2024 Mar 28;15:1360540. doi: 10.3389/fpsyg.2024.1360540 (PMC11007186; doi:10.3389/fpsyg.2024.1360540)
Supplement: Supplementary file 1 [file Table_1.DOCX]

**Supplementary Table 1 ⎜**Search strategy for APA PsycInfo (EBSCOhost).

| S1 | AB (impost?r N2 (phenomenon* OR syndrome* OR experience*)) OR "fraud syndrom*" OR impostorism |
| --- | --- |
| S2 | TI (impost?r N2 (phenomenon* OR syndrome* OR experience*)) OR "fraud syndrom*" OR impostorism |
| S3 | S1 OR S2 |
| S4 | AB therap* OR treatment* OR intervention* OR counseling OR management OR rehabilitation* |
| S5 | DE "Counseling" OR DE "Management" OR DE "Intervention" OR DE "Treatment" OR DE "Rehabilitation" |
| S6 | S4 OR S5 |
| S7 | S3 AND S6 |
